# Optimizing adrenal vein sampling in primary aldosteronism subtyping through LC–MS/MS and secretion ratios of aldosterone, 18-oxocortisol, and 18-hydroxycortisol

**DOI:** 10.1038/s41440-023-01347-2

**Published:** 2023-06-13

**Authors:** Yu-Ling Chang, Guan-Yuan Chen, Bo-Ching Lee, Po-Ting Chen, Kao-Lang Liu, Chin-Chen Chang, Te-I Weng, Vin-Cent Wu, Yen-Hung Lin, the TAIPAI Study Group

**Affiliations:** 1 Department of Medical Imaging, National Taiwan University Hospital and National Taiwan University College of Medicine, Taipei, Taiwan; 2 Department and Graduate Institute of Forensic Medicine, National Taiwan University College of Medicine, Taipei, Taiwan; 3 Department of Internal Medicine, National Taiwan University Hospital and National Taiwan University College of Medicine, Taipei, Taiwan

**Keywords:** Adrenal glands, Aldosterone, Hyperaldosteronism, Mass spectrometry

## Abstract

Adrenal venous sampling (AVS) is the gold standard for identifying curable unilateral aldosterone excess in primary aldosteronism (PA). Studies have demonstrated the value of steroid profiling through liquid chromatography–tandem mass spectrometry (LC–MS/MS) in AVS interpretation. First, the performance of LC–MS/MS and immunoassay in assessing selectivity and lateralization was compared. Second, the utility of the proportion of individual steroids in adrenal veins in subtyping PA was analyzed. We enrolled 75 consecutive patients with PA who underwent AVS between 2020 and 2021. Fifteen adrenal steroids were analyzed in peripheral and adrenal veins through LC–MS/MS before and after adrenocorticotropic hormone (ACTH) stimulation. Through selectivity index that was based on cortisol and alternative steroids, LC–MS/MS rescued 45% and 66% of failed cases judged by immunoassay in unstimulated and stimulated AVS, respectively. LC–MS/MS identified more unilateral diseases than did immunoassay (76% vs. 45%, *P* < 0.05) and provided adrenalectomy opportunities to 69% of patients judged through immunoassay to have bilateral disease. The secretion ratios (individual steroid concentration/total steroid concentration) of aldosterone, 18-oxocortisol, and 18-hydroxycortisol were novel indicators for identifying unilateral PA. The 18-oxocortisol secretion ratio of ≥0.785‰ (sensitivity/specificity: 0.90/0.77) at pre-ACTH and aldosterone secretion ratio of ≤0.637‰ (sensitivity/specificity: 0.88/0.85) at post-ACTH enabled optimal accuracy for predicting ipsilateral and contralateral disease, respectively, in robust unilateral PA. LC–MS/MS improved the success rate of AVS and identified more unilateral diseases than immunoassay. The secretion ratios of steroids can be used to discriminate the broad PA spectrum.

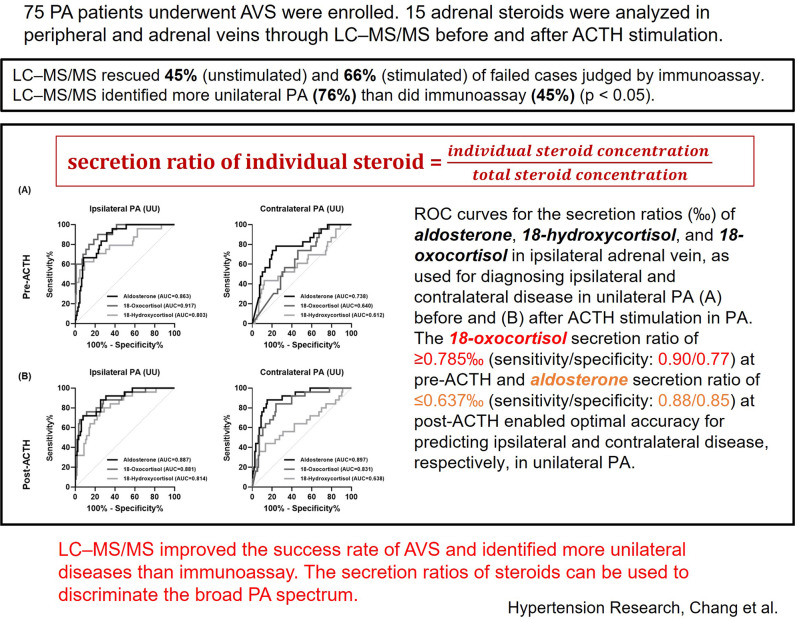

## Introduction

Primary aldosteronism (PA) is the most common cause of secondary hypertension, and it is surgically curable if unilateral excessive aldosterone secretion is identified. Adrenal venous sampling (AVS) is the gold standard for distinguishing unilateral aldosterone sources from bilateral ones in PA. However, only a minority of patients with PA who undergo AVS are eventually referred for unilateral adrenalectomy [1]. In addition to the complexity of AVS and the variable interpretation criteria, the use of cortisol as an indicator can lead to inclusive findings in successful catheterization and lateralization assessments because cortisol has a long half-life and undergoes physical fluctuations [2]. Fortunately, with advances in liquid chromatography–tandem mass spectrometry (LC–MS/MS), recent studies have revealed the utility of alternative steroids in AVS interpretation, including 11-deoxycortisol and corticosterone in selectivity index (SI) assessments [3] and 18-oxocortisol and 18-hydroxycortisol in lateralization index (LI) assessments [2].

Nevertheless, research on the overall performance of LC–MS/MS-derived SI and LI relative to immunoassay remains limited. Herein, we compared LC–MS/MS and immunoassay in terms of their AVS success rates and lateralization results. In addition, LC–MS/MS can be used to measure the ratio of individual steroid concentration to total steroid concentration in a single adrenal vein (AV). Therefore, our second goal was to establish indicators for predicting lateralization accurately by using unilateral AV samples.

## Materials and methods

### Study population

We retrospectively enrolled 80 patients with clinically suspected PA who underwent AVS between May 2020 and May 2021 and were followed up until February 2022 at National Taiwan University Hospital. We used prospectively collected data from the Taiwan Primary Aldosteronism Investigators (TAIPAI) database to standardize data collection [4]. Five patients were excluded from the study. Specifically, one patient failed to meet the diagnostic criteria for PA, three had overt Cushing syndrome, and one had congenital adrenal hyperplasia. All patients with confirmed PA underwent an overnight 1-mg dexamethasone suppression test (DST), adrenal computed tomography (CT) scanning, and LC–MS/MS‐based steroid profiling during AVS. All the participants in the present study provided written informed consent, and the study protocol was approved by an institutional review board.

### Diagnosis of PA and autonomous cortisol secretion

PA screening, confirmation, and subtype differentiation were performed in accordance with the aforementioned TAIPAI protocol [4]. In brief, we used the plasma aldosterone concentration (PAC)/plasma renin activity (PRA) ratio (ARR) to detect possible cases of PA. A PA diagnosis was confirmed through a saline infusion test or captopril challenge test following a positive screening. Antihypertensive and interfering medication were discontinued for all participating patients, except those with severe hypertension, who were switched to calcium-channel blockers or α-blockers until final diagnosis.

Because Asian patients with PA have a high prevalence of autonomous cortisol secretion (ACS) [5], a low-dose (1 mg) DST was conducted in all patients with suspected PA for the ACS screening process. Specifically, 1 mg of dexamethasone was administered at 2300 h on the first day of the test, and a cortisol measurement was taken at 0800 h on the following day. Although standards for adequate cortisol suppression for DSTs have yet to be established, the European Endocrinology Society proposed a cutoff point of 1.8 μg/dL to rule out ACS [6]. However, a study used a lower cutoff value to predict a lower clinical complete success rate for patients with aldosterone-producing adenoma (APA) [7]. Therefore, we used a cutoff value of 1.8 μg/dL in the present study. We measured cortisol, aldosterone, and PRA with immunoassays in accordance with a previous report [7].

### AVS and data interpretation

AVS was performed by a single radiologist with more than 10 years of experience in interventional vascular procedures. Blood samples were obtained simultaneously from bilateral AVs and the inferior vena cava (IVC). After unstimulated AVS was conducted, ACTH‐stimulated AVS was performed (sampling was performed for 15 min after an intravenous bolus injection of 0.25 mg cosyntropin). Catheterization was regarded to be successful when the SI (defined by AV/IVC cortisol concentrations) was ≥2 before ACTH stimulation and ≥5 after ACTH stimulation. The lateralization of PA was assessed through the LI (defined as the aldosterone/cortisol ratio between the dominant and nondominant AV). Unilateral PA was diagnosed if the LI was ≥2 before ACTH stimulation and ≥4 after ACTH stimulation. Contralateral suppression was indicated by a contralateral suppression index (defined as the aldosterone/cortisol ratio between the nondominant AV and IVC) of <1.

### Immunoassay measurement

Serum cortisol was measured using a chemiluminescent microparticle immunoassay (Architect, Abbott, VA, USA). For cortisol, the intraassay and interassay coefficients of variation (CVs) were 3.3% and 3.4%, respectively, whereas the limit of detection (LOD) was 8 ng/mL. Plasma aldosterone was measured using radioimmunoassay kits in accordance with the manufacturer’s instructions (ALDO-RIACT, Cisbio Bioassays, Codolet, France). For aldosterone, the intraassay and interassay CVs were 7.7% and 8.4%, respectively, whereas the LOD was 0.007 ng/mL.

### LC–MS/MS-based steroid profiling

We measured 15 types of adrenal steroids in a single assay through LC–MS/MS (an Agilent 1290 UHPLC coupled to a Sciex 6500 Qtrap); the measured steroids were as follows: aldosterone, cortisol, androstenedione, corticosterone, cortisone, 11-deoxycortisol, 21- deoxycortisol, 18-hydroxycortisol, 18-oxocortisol, dehydroepiandrosterone (DHEA), DHEA-sulfate (DHEAS), 17-hydroxyprogesterone, progesterone, testosterone, and tetrahydrocorticosterone. Serum steroids were extracted and analyzed in accordance with the protocol used in a previous report [8]. In brief, a 100-µL serum sample was diluted with 900-µL deionized water, after which a solid-phase extraction was performed in accordance with the manufacturer’s instructions. Isotope-coded steroid standards were employed for LC–MS/MS quantification. The limit of quantification for steroid hormones was between 0.05 and 60 ng/mL, which was based on the properties of the tested steroid hormones. Additional detailed descriptions of materials and methods are available in the Supplementary information (Supplemental Methods and Supplementary Table S1) [9].

### Follow-up and outcome evaluation

The Primary Aldosteronism Surgery Outcome consensus on clinical and biochemical outcomes was applied for patients with PA who underwent adrenalectomy [10].

### Statistical analysis

Unless otherwise specified, all data are expressed as means ± standard deviations or median and interquartile ranges. A Deming regression analysis and Bland–Altman plots were used to compare the results of immunoassay and LC–MS/MS. Normality and data variance were examined using the Shapiro–Wilk normality test. Relationships were assessed using the Spearman correlation coefficient (*r*_*s*_). Statistical analyses were performed using the one-sample *t*-test for continuous variables, Mann–Whitney *U* test, Kruskal–Wallis test (for nonparametric variables), and *χ*^2^ test (for categorical variables). Paired variables were assessed using the Wilcoxon signed-rank test and McNemar’s test. A receiver operator characteristic (ROC) analysis was used to determine the optimal variable cutoff values for predicting PA lateralization. All analyses were conducted using the SPSS version 25 (IBM, Armonk, NY, USA). All figures were created using the GraphPad Prism 9 software. For all statistical analyses, significance was indicated by a two-sided *P* value of <0.05.

## Results

### Baseline characteristics

Because the influence of ACS on the AVS interpretation of catheterization success and lateralization in unstimulated AVS is unclear [5], patients with and without ACS were analyzed separately. Of the 75 patients with PA, ACS was suggested for 13 (17.3%) patients after they underwent the 1-mg DST. No significant intergroup differences were observed for baseline characteristics. Both the ACS and non-ACS groups exhibited the biochemical features that were expected for a PA cohort (high PAC and ARR with low PRA). Table 1 summarizes the related clinical and biochemical data.

**Table 1:** Clinical and biochemical parameters at diagnosis

	Total	PA without ACS	PA with ACS	*P*
*N*	75	62	13	–
Sex, Female	54.1%	58.1%	30.8%	NS
Age, years	55.0 ± 11.5	54.9 ± 11.8	55.6 ± 10.6	NS
SBP, mm Hg	154.9 ± 24.8	155.9 ± 24.6	150.3 ± 26.5	NS
DBP, mm Hg	91.7 ± 14.4	93.1 ± 14.8	87.9 ± 10.4	NS
Potassium, mmol/L	3.57 ± 0.58	3.56 ± 0.80	3.62 ± 0.58	NS
Post-ONDST cortisol, μg/dL	1.10 (1.00–1.70)	1.00 (1.00–1.10)	3.40 (2.33–5.03)	<0.0001
PAC, ng/dL	26.9 (18.1–39.1)	31.4 (20.8–40.4)	20.0 (18.2–22.4)	NS
PRA, ng/mL/h	0.47 (0.23–1.30)	0.40 (0.20–1.37)	0.93 (0.47–1.35)	NS
ARR	50.4 (22.2–119.7)	60.7 (23.9–131.1)	22.3 (16.1–30.6)	NS

*χ*^2^ test for categorical variables, one-sample *t*-test for continuous variables, and Mann–Whitney *U* test for nonparametric variables. Statistical significance was set at *P* < 0.05. Values are expressed as means ± standard deviations or medians (interquartile ranges)

*ACS* autonomous cortisol secretion, *ARR* aldosterone-renin ratio, *DBP* diastolic blood pressure, *NS* not significant, *ONDST* overnight (1 mg) dexamethasone suppression test, *PA* primary aldosteronism, *PAC* plasma aldosterone concentration, *PRA* plasma renin activity, *SBP* systolic blood pressure

### Immunoassay and LC–MS/MS comparisons

Strong positive relationships were observed between measurements of cortisol (*r*_*s*_ = 0.962, *P* < 0.0001) by immunoassay and LC–MS/MS and moderated positive relationships were observed between measurements of aldosterone (*r*_*s*_ = 0.5306, *P* < 0.0001) by immunoassay and LC–MS/MS. The Deming regression revealed a more favorable agreement for measurements of cortisol between the two methods. Overall, plasma cortisol and aldosterone were higher when LC–MS/MS was used than when immunoassay was used, and a significant difference (*P* < 0.001) between the peripheral and AVs was observed (Supplementary Fig. S1) [9].

### LC–MS/MS-derived SI and success rate

The overall success rate of bilateral AV cannulation using cortisol-based SI was higher when LC–MS/MS was used than when immunoassay was used for both the unstimulated AVS (85.3% vs. 80%) and stimulated AVS (88% vs. 85.3%). We further analyzed 13 types of alternative steroids and discovered that five types of steroids (androstenedione, corticosterone, 11-deoxycortisol, 17-hydroxyprogesterone, and DHEA) had higher SIs than cortisol-based SIs when immunoassay was used; on average, they were between 1.8- and 2.3-fold greater and between 1.5- and 5.1- fold greater before and after ACTH stimulation, respectively (all *P* < 0.05). In addition, progesterone-based SI was 2.2-fold higher in stimulated AVS (*P* < 0.05; Supplementary Fig. S2) than in unstimulated AVS [9]. When the aforementioned alternative steroids were used as indicators of selectivity, 45% and 66% of the failed cases were rescued in unstimulated AVS and stimulated AVS, respectively. Overall, 69 (92%) and 71 (94.6%) patients were regarded to have undergone successful bilateral AV cannulation at pre-ACTH and post-ACTH on the basis of the LC–MS/MS-derived SIs that were based on cortisol and alternative steroids. No statistical difference in success rate was detected between the ACS and non-ACS groups.

### LC–MS/MS-derived LI and PA lateralization

**Fig. 1:**
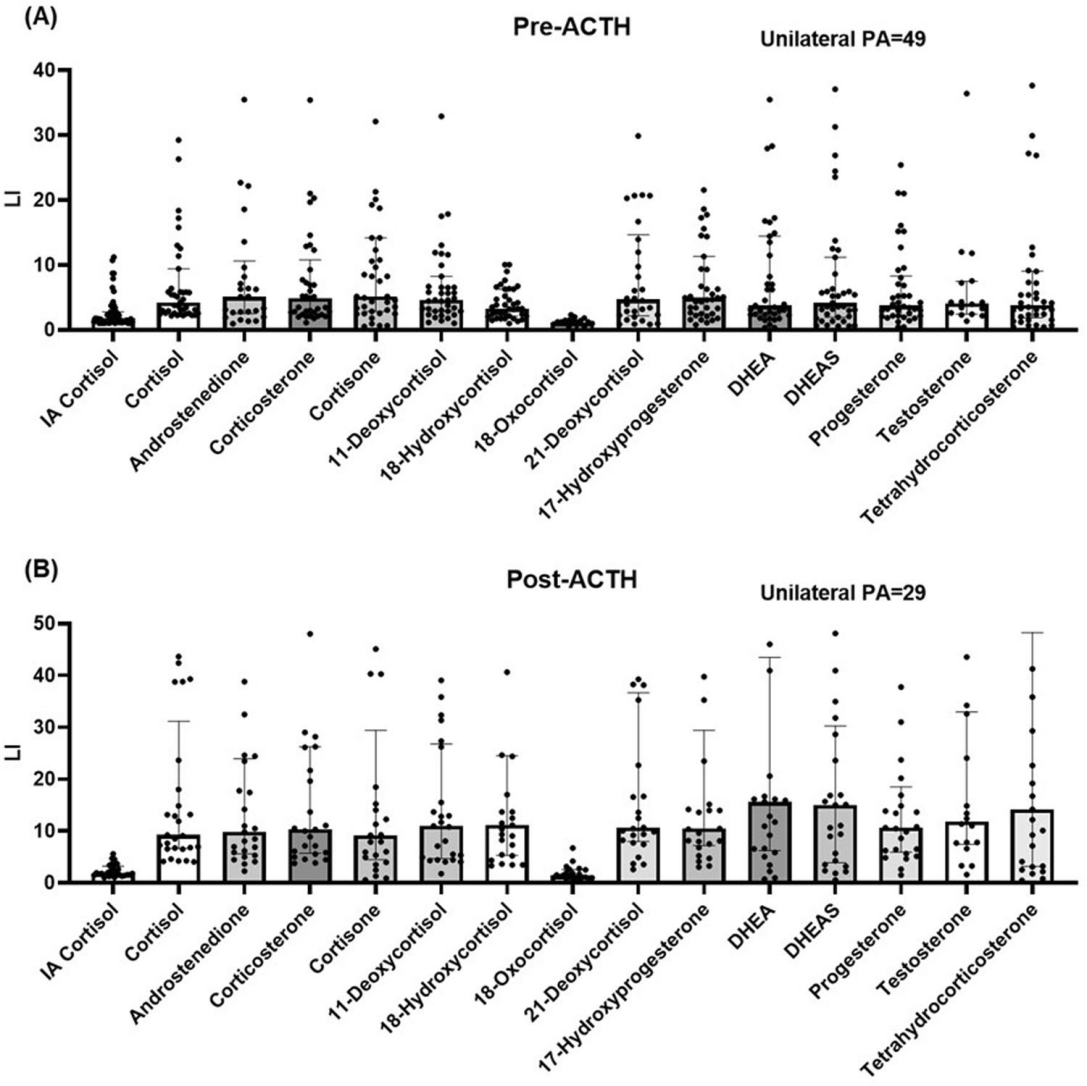
Comparison of LIs normalized by immunoassay cortisol and LC–MS/MS steroids in unilateral primary aldosteronism that was classified by LC–MS/MS results at **A** pre-ACTH and **B** post-ACTH stimulation AVS. All LC–MS/MS steroid-normalized LIs (except for that normalized by 18-oxocortisol) were higher than the immunoassay cortisol-normalized LIs (all *P* < 0.01). A pairwise within-patient comparison was performed using a Wilcoxon signed-rank test. The boxes represent the median, and the horizontal line marks the interquartile range. ACTH adrenocorticotropic hormone, IA immunoassay, LC–MS/MS liquid chromatography–tandem mass spectrometry, LI lateralization index, PA primary aldosteronism

We used 13 types of alternative steroids to normalize the aldosterone level in LC–MS/MS-derived LIs. Except for 18-hydroxycortisol and 18-oxocortisol, the other 11 steroid-normalized LIs exhibited a moderate to strong correlation with cortisol-normalized LIs (*r*_*s*_ > 0.65, all *P* < 0.01) for both unstimulated and stimulated AVS. A pairwise within-patient comparison (for the 71 patients who underwent successful AVS) revealed that except for 18-oxocortisol, the LC–MS/MS-derived LIs normalized by cortisol and 12 alternative steroids were significantly higher (all *P* < 0.001) than the immunoassay-derived LIs. The difference was more pronounced (*P* < 0.001) in the unilateral PA that was classified on the basis of LC–MS/MS lateralization results (Fig. 1). Among the patients with unilateral PA, their LIs were between 47% and 91% higher in unstimulated AVS and between 109% and 144% higher in stimulated AVS when LC–MS/MS was used than when immunoassay was used. No difference in LC–MS/MS-derived LI was detected between the ACS and non-ACS groups.

**Fig. 2:**
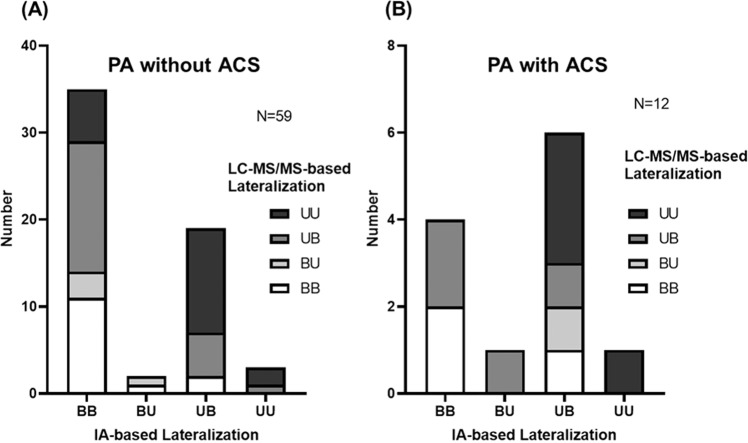
Number of patients with PA in **A** non-ACS group and **B** ACS group of various LC–MS/MS lateralization groups that were stratified by immunoassay lateralization. PA lateralization was classified into four categories according to LI pre-ACTH and post-ACTH; the four categories are concordant results with bilateral PA (BB) at pre-ACTH and post-ACTH, concordant results with unilateral PA (UU) at pre-ACTH and post-ACTH, discrepant results with unilateral PA only at pre-ACTH (UB), and discrepant results with unilateral PA only at post-ACTH (BU). Through LC–MS/MS, lateralization was revealed to be concordant in 41 patients (non-ACS/ACS: UU = 20/4, BB = 14/3) and discordant in 30 patients (non-ACS/ACS: UB = 21/4, BU = 4/1). Through immunoassay, lateralization was revealed to be concordant in 43 patients (non-ACS/ACS: UU = 3/1, BB = 35/4) and discordant in 28 patients (non-ACS/ACS: UB = 19/6, BU = 2/1). ACS autonomous cortisol secretion, IA immunoassay, LC–MS/MS liquid chromatography–tandem mass spectrometry, PA primary aldosteronism

Because of the LI differences between LC–MS/MS and immunoassay, a PA lateralization indicated discordance between assays (Fig. 2). We classified PA lateralization status into four categories according to the LI before and after ACTH stimulation; the four categories are concordant bilateral PA (BB) at pre-ACTH and post-ACTH, unilateral PA (UU) at pre-ACTH and post-ACTH, discrepant results with unilateral PA detected only at pre-ACTH (UB), and discrepant results with unilateral PA detected only at post-ACTH (BU). In the patients without ACS, 68.6% bilateral PA (BB) based on immunoassay became unilateral PA (UB, BU, or UU) when LC–MS/MS LI normalized by cortisol was used (*P* < 0.0001). Furthermore, 57.1% of the patients with discrepant lateralization results (UB or BU) based on immunoassay were determined to have robust unilateral PA (UU) when LC–MS/MS was used (*P* < 0.0001). We observed a similar trend in patients with ACS. Overall, 54 patients (76%) and 32 patients (45%) were determined to have unilateral diseases (UB, BU, and UU) when LC–MS/MS and immunoassay, respectively, were used.

### Postoperative outcome

In total, 27 patients underwent unilateral adrenalectomy, and 20 cases of cortical adenoma and 7 cases of adrenal hyperplasia were confirmed through histological analysis. In addition, we assessed the postoperative outcomes of 11 patients who were followed up for at least 1 year. All of these patients achieved complete biochemical success, with 63.6% and 36.3% achieving complete and partial clinical success, respectively. For these 11 patients, two assays revealed concordant unilateral PA results for nine patients but discordant results for two patients. These two patients had unilateral PA according to LC–MS/MS and bilateral PA according to immunoassay, and both achieved the complete clinical and biochemical resolution of their PA.

### Comparison of secretion ratio of steroids in PA subtypes

The secretion ratio of steroids is the ratio of individual steroid concentration to total steroid concentration in the unilateral AV (secretion ratio of individual steroid = $\frac{{{{{{{\rm{individual}}}}}}\; {{{{{\rm{steroid}}}}}}\; {{{{{\rm{concentration}}}}}}}}{{{{{{{\rm{total}}}}}}\; {{{{{\rm{steroid}}}}}}\; {{{{{\rm{concentration}}}}}}}}$) [11]. Through LC–MS/MS, we compared the secretion ratios of steroids in dominant and nondominant AVs among the lateralization groups (Table 2). In patients with robust unilateral disease (UU), the secretion ratios of aldosterone, 18-hydroxycortisol, and 18-oxocortisol were higher in dominant AVs than in nondominant AVs (all *P* < 0.0001). In patients with unilateral results only at pre-ACTH (UB), the secretion ratios of aldosterone and 18-oxocortisol were significantly higher in dominant AVs than in nondominant AVs (all *P* < 0.01). By contrast, in patients with robust bilateral disease (BB), the secretion ratios of all 15 steroids were not significantly different among bilateral AVs.

**Table 2:** Comparison of steroid secretion ratios (‰) of dominant and nondominant adrenal veins by PA lateralization status at pre-ACTH and post-ACTH stimulation

	Unilateral PA (UU = 24)			Unilateral PA (UB = 25)			Bilateral PA (BB = 17)		
	Dominant AV	Nondominant AV	*P*	Dominant AV	Nondominant AV	*P*	Dominant AV	Nondominant AV	*P*
*Pre-ACTH*									
Aldosterone	7.78 (2.61–11.3)	0.25 (0.01–0.64)	<0.0001	1.34 (0.92–3.05)	0.35 (0.01–0.83)	<0.0001	1.98 (0.95–7.52)	1.15 (0.79–5.13)	NS
Cortisol	534 (425–724)	605 (413–745)	NS	571 (450–667)	605 (459–698)	NS	527 (330–691)	606 (299–689)	NS
Androstenedione	0.38 (0.01–13.8)	0.38 (0.04–13.3)	NS	0.48 (0.01–14.9)	0.58 (0.09–15.9)	NS	8.86 (1.06–20.0)	11.8 (0.51–23.7)	NS
Corticosterone	40.8 (24.9–60.1)	31.2 (19.8–53.7)	NS	32.1 (17.1–56.7)	27.9 (19.6–47.5)	NS	21.9 (16.3–40.8)	28.8 (16.8–45.9)	NS
Cortisone	30.6 (22.3–54.2)	36.1 (21.4–47.8)	NS	39.3 (27–52.8)	37.8 (30.8–50.0)	NS	34.4 (26.9–42.9)	32.0 (23.7–43.2)	NS
11-Deoxycortisol	9.83 (7.31–16.2)	11.1 (4.83–14.2)	NS	8.50 (5.42–11.2)	7.10 (5.30–11.9)	NS	6.32 (3.62–11.3)	6.30 (3.71–10.2)	NS
18-Hydroxycortisol	12.5 (5.76–21.5)	4.01 (2.24–7.85)	<0.0001	4.74 (3.72–6.93)	4.66 (3.41–6.09)	NS	5.07 (3.03–6.09)	4.23 (2.53–6.22)	NS
18-Oxocortisol	3.38 (0.54–6.27)	0.17 (0.01–0.58)	<0.0001	0.23 (0.01–0.68)	0.01 (0.01–0.21)	0.005	0.69 (0.47–1.73)	0.27 (0.05–0.96)	NS
21-Deoxycortisol	1.08 (0.59–2.18)	0.85 (0.48–2.03)	NS	1.30 (0.51–1.97)	0.95 (0.01–2.28)	NS	1.24 (0.01–4.02)	1.26 (0.28–7.26)	NS
17-Hydroxyprogesterone	8.94 (5.03–13.1)	6.78 (4.64–12.2)	NS	6.25 (4.22–11.8)	5.40 (4.13–12.6)	NS	6.28 (2.73–11.0)	7.94 (3.63–18.4)	NS
DHEA	7.33 (4.29–12.9)	7.98 (2.82–16.9)	NS	8.52 (4.77–11.8)	8.48 (3.82–12.0)	NS	6.55 (4.04–14.6)	9.02 (4.29–15.0)	NS
DHEAS	276 (95.6–434)	204 (73.8–376)	NS	282 (129–424)	258 (111–440)	NS	326 (93.2–561)	213 (102–537)	NS
Progesterone	3.58 (1.91–6.23)	2.07 (0.74–3.84)	NS	1.63 (0.95–4.15)	1.47 (0.85–3.23)	NS	1.52 (0.76–3.06)	2.89 (0.73–6.29)	NS
Testosterone	0.01 (0.01–0.17)	0.01 (0.01–0.12)	NS	0.01 (0.01–0.08)	0.01 (0.01–0.10)	NS	0.07 (0.01–0.16)	0.10 (0.03–0.17)	NS
Tetrahydrocorticosterone	9.74 (4.92–29.4)	10.2 (5.68–23.2)	NS	9.43 (5.58–21.6)	8.64 (5.53–18.3)	NS	6.57 (3.79–16.4)	6.87 (3.83–24.4)	NS
*Post-ACTH*									
Aldosterone	5.21 (2.33–9.41)	0.29 (0.13–0.52)	<0.0001	1.43 (0.89–2.75)	0.71 (0.49–1.41)	0.003	2.65(1.6–3.17)	1.83 (0.94–2.31)	NS
Cortisol	593 (549–665)	645 (563–730)	NS	637 (608–694)	632 (575–685)	NS	634 (589–731)	670 (610–718)	NS
Androstenedione	0.45 (0.29–9.06)	0.33 (0.26–9.03)	NS	0.37 (0.22–14.5)	0.34 (0.2–13.65)	NS	10.9 (0.24–18.8)	12.4 (0.27–20.6)	NS
Corticosterone	140 (106–214)	132 (98.5–172.7)	NS	137 (104–155)	131 (90.3–148)	NS	113 (77.6–135)	106 (87.3–138)	NS
Cortisone	20.2 (16.9–31.6)	22.4 (17.4–30.9)	NS	29.1 (21.2–34.3)	26.3 (20–34.3)	NS	23.9 (19.5–29.8)	21.2(18.6–29.1)	NS
11-Deoxycortisol	18.7 (13.6–25.4)	15.1 (11.2–20)	0.035	15.6 (12.9–20.9)	14.4 (10.8–22.7)	NS	15.3 (12.9–17.5)	16.7 (11.1–18.7)	NS
18-Hydroxycortisol	6.03 (4.67–8.65)	3.21 (2.11–5.15)	<0.0001	4.08 (3.15–5.32)	3.53 (2.72–5.3)	NS	3.52 (2.86–4.28)	3.46 (2.53–4.21)	NS
18-Oxocortisol	1.17 (0.56–2.08)	0.13 (0.08–0.16)	<0.0001	0.27 (0.19–0.52)	0.15 (0.11–0.25)	0.008	0.45 (0.21–0.64)	0.28 (0.17–0.56)	NS
21-Deoxycortisol	3.16 (1.32–5.79)	2.93 (1.67–8.35)	NS	3.35 (2.1–5.57)	3.53 (2.16–5.74)	NS	3.52 (2.33–7.91)	3.93 (2.62–11.1)	NS
17-Hydroxyprogesterone	22.3 (14.7–41.7)	22.9 (15.5–43.7)	NS	27.4 (19.6–42.6)	24.2 (17.3–44.1)	NS	29.3 (20.2–36.2)	36.0 (22.4–50.0)	NS
DHEA	9.35 (5.24–13.3)	9.62 (5.25–16.9)	NS	9.96 (7.4–13.5)	8.23 (5.31–13.2)	NS	9.97 (6.19–16.9)	11.7 (6.73–19.1)	NS
DHEAS	77.4 (30.4–120)	58.8 (27.1–157)	NS	97.5 (42.9–144)	81.7 (29.2–129)	NS	103 (32.1–167)	71.9 (36.9–115)	NS
Progesterone	21.6 (13.3–30.3)	18.1 (9.74–36.9)	NS	18.7 (10.3–26.1)	14.5 (8.69–29.5)	NS	14.5 (9.58–19.4)	14.5 (10.8–20.8)	NS
Testosterone	0.07 (0.04–0.13)	0.08 (0.04–0.13)	NS	0.06 (0–0.07)	0.06 (0.01–0.07)	NS	0.06 (0.01–0.12)	0.07 (0.04–0.11)	NS
Tetrahydrocorticosterone	6.43 (3.09–9.83)	6.86 (4.83–9.42)	NS	6.52 (5.72–11.6)	6.06 (4.35–8.51)	NS	7.54 (5.77–9.44)	8.34 (5.62–10.6)	NS

Steroid secretion ratio (‰) was calculated by dividing the amount of each steroid by the total amount of steroids. Differences were evaluated using the Mann–Whitney *U* test. Statistical significance was set at *P* < 0.05. Values are expressed as medians (interquartile ranges). PA lateralization status was classified into four categories according to LI pre-ACTH and post-ACTH; the four categories are concordant bilateral PA (BB) at pre-ACTH and post-ACTH, unilateral PA (UU) at pre-ACTH and post-ACTH (UU), discrepant results with unilateral PA detected only at pre-ACTH (UB), discrepant results with unilateral PA detected only at post-ACTH (BU). The subgroup of BU is not shown in the table because only a small sample was used (*n* = 5)

*ACTH* adrenocorticotropic hormone, *AV* adrenal vein, *NS* not significant, *PA* primary aldosteronism

### Unilateral secretion ratio of steroids in PA subtypes prediction

**Fig. 3:**
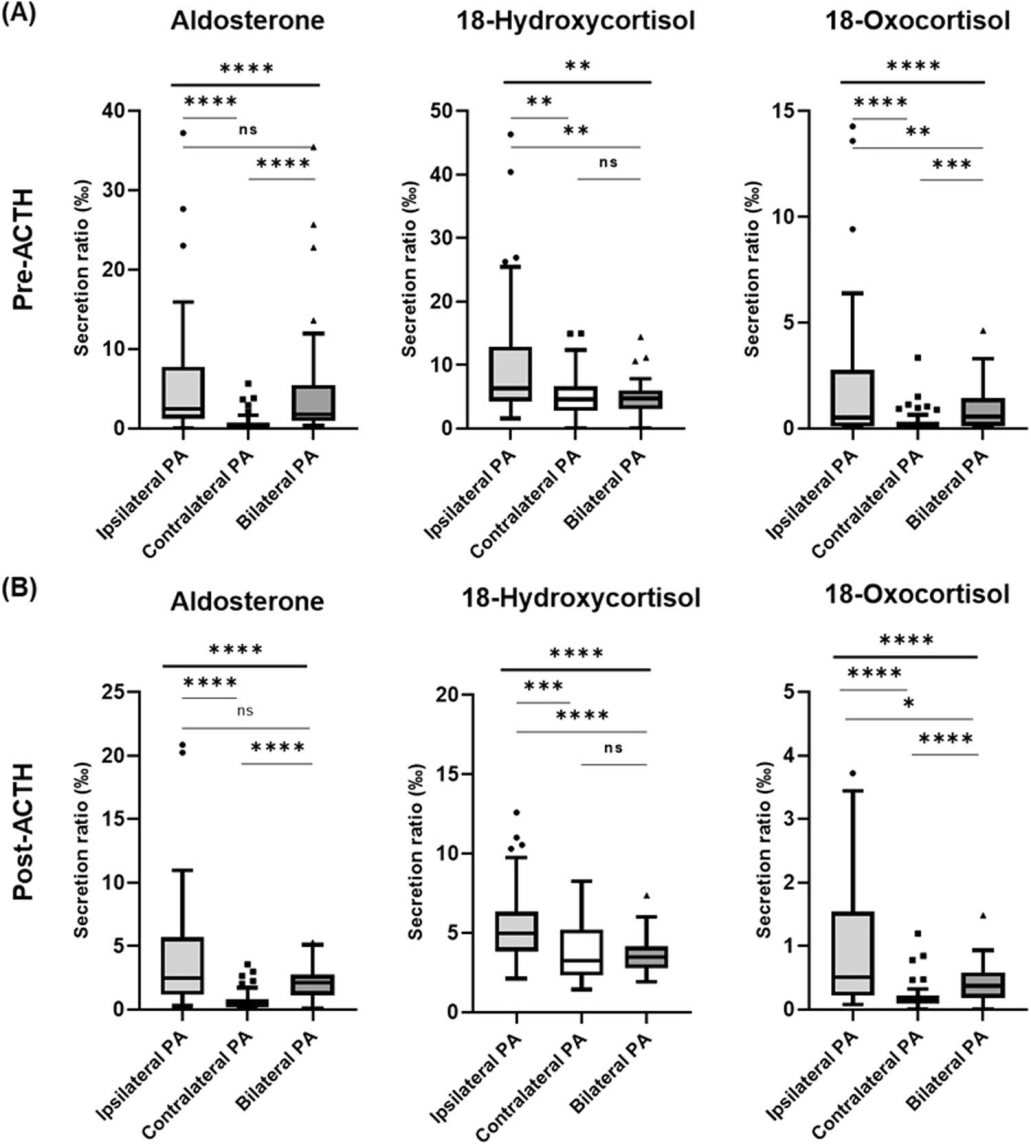
Tukey box–whisker plot comparison of the secretion ratios (‰) of aldosterone, 18-hydroxycortisol, and 18-oxocortisol in the unilateral AV of various lateralization statuses (classified by LS–MS/MS results) in patients with PA **A** before and **B** after ACTH stimulation. The secretion ratio (‰) of steroids was calculated by dividing the amount of each steroid by the total amount of steroids in unilateral AV. Intergroup differences were evaluated with the Kruskal–Wallis test (*P < 0.05, **P < 0.01, ***P < 0.00, ****P < 0.0001 between groups). ACS autonomous cortisol secretion, ACTH adrenocorticotropic hormone, AV adrenal vein, PA primary aldosteronism

We compared the unilateral secretion ratios of aldosterone, 18-hydroxycortisol, and 18-oxocortisol in patients with unilateral ipsilateral disease, unilateral contralateral disease, and bilateral disease (Fig. 3). The term “ipsilateral” and “contralateral” disease referred to the assessment of whether the tested unilateral adrenal hormone is localized on the same side or opposite side, based on pre- and post-ACTH AVS as the reference standard. First, the secretion ratios of all three steroids were, as predicted, significantly higher in patients with unilateral ipsilateral disease than in those with contralateral disease. Furthermore, the secretion ratios of 18-hydroxycortisol and 18-oxocortisol were significantly higher in patients with unilateral ipsilateral disease than in those with bilateral disease. Third, the secretion ratios of aldosterone and 18-oxocortisol were significantly lower in patients with unilateral contralateral disease than in those with bilateral disease. The aforementioned results were all statistically significant both before and after ACTH stimulation.

**Fig. 4:**
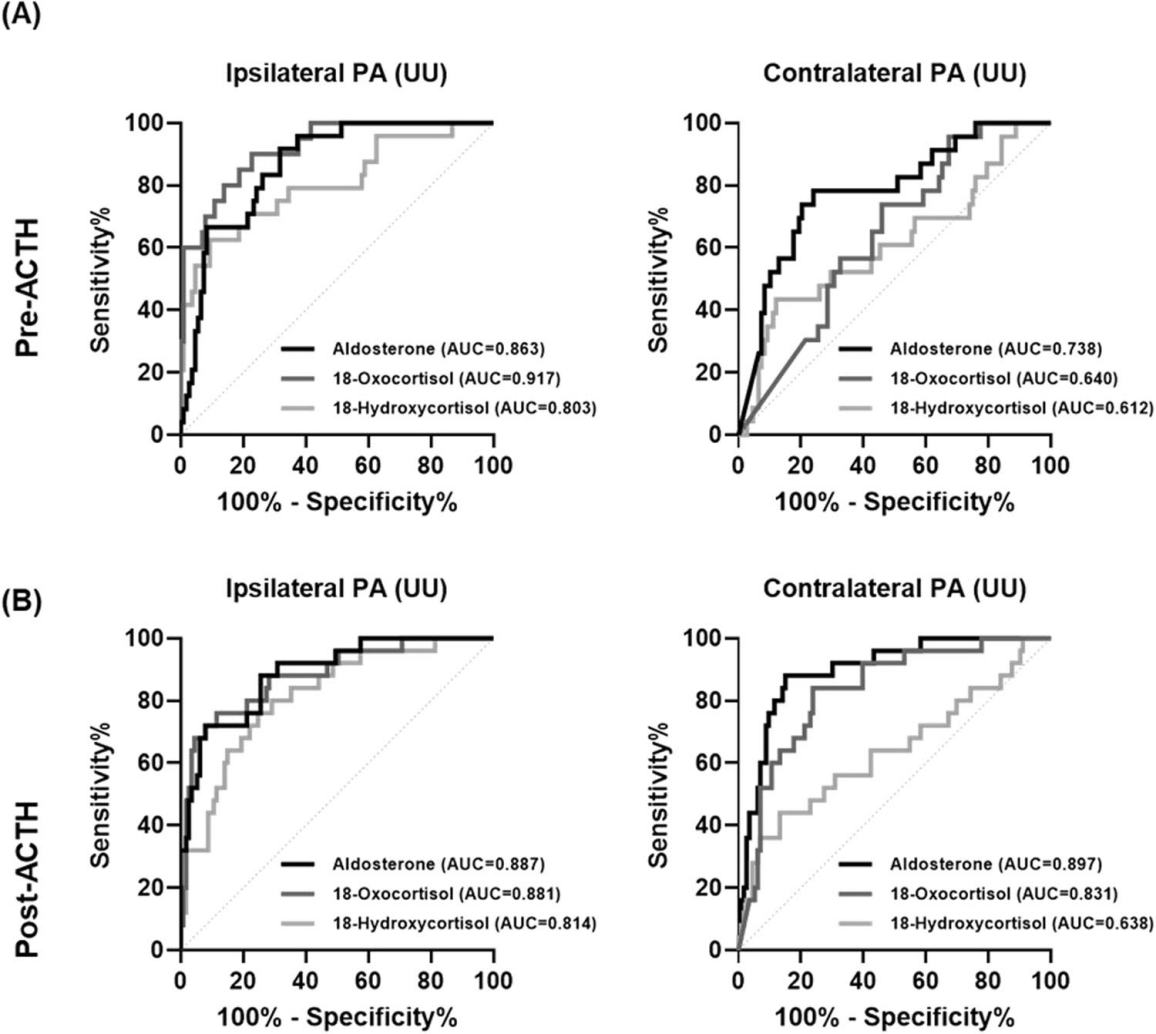
ROC curves for the secretion ratios (‰) of aldosterone, 18-hydroxycortisol, and 18-oxocortisol, as used for diagnosing ipsilateral disease and contralateral disease in robust unilateral PA **A** before and **B** after ACTH stimulation in primary aldosteronism (PA). UU indicates robust unilateral PA with concordant results in unstimulated and stimulated AVS. The secretion ratio of steroids was calculated by dividing the amount of each steroid by the total amount of steroids in the unilateral AV. ACTH adrenocorticotropic hormone, AV adrenal vein, AVS adrenal venous sampling, ROC receiver operator characteristic, PA primary aldosteronism

The ROC curves indicated the utility of unilateral aldosterone, 18-hydroxycortisol, and 18-oxocortisol secretion ratios for the diagnosis of robust unilateral PA (UU) (Fig. 4). The optimal cutoff points were obtained using the ROC curves and Youden’s Index (Table 3). In unstimulated AVS, the optimal cutoff point for predicting unilateral ipsilateral diseases was ≥0.785 (‰) when the 18-oxocortisol secretion ratio was applied, with an area under the curve (AUC) of 0.92. In stimulated AVS, the optimal cutoff points for predicting unilateral ipsilateral and contralateral diseases were ≥3.352 (‰) and ≤0.637 (‰) when the aldosterone secretion ratio was applied, with AUCs of 0.89 and 0.90, respectively. However, the secretion ratios had limited value in predicting contralateral diseases before ACTH stimulation. The application of an aldosterone secretion ratio of ≤0.637 following ACTH stimulation led to the most favorable diagnostic results for unilateral PA in our study, that is, a 95.8% accuracy for robust unilateral PA and 53% accuracy for intermediate unilateral PA; however, 11.8% (2/17) of the patients were misclassified.

**Table 3:** Optimal cutoff values of secretion ratios (‰) of aldosterone, 18-hydroxycortisol, and 18-oxocortisol for prediction of unilateral PA (UU) in our cohort (*n* = 71 with successful AVS)

Diagnosing for		AUC (95% CI)	Cutoffs (‰)	Sensitivity (95% CI)	Specificity (95% CI)
	*Pre-ACTH*				
Ipsilateral PA (UU)	Aldosterone	0.86 (0.79–0.93)	>1.664	91.7 (74.2–98.5)	68.2 (58.9–76.3)
	18-Oxocortisol	0.92 (0.86–0.98)	>0.785	90.0 (69.9–98.2)	77.2 (68.1–84.3)
	18-Hydroxycortisol	0.80 (0.69–0.91)	>9.927	62.5 (42.7–78.8)	90.7 (83.7–94.8)
Contralateral PA (UU)	Aldosterone	0.78 (0.68–0.89)	<0.675	78.3 (58.1–90.3)	75.9 (67.1–83.0)
	18-Oxocortisol	0.64 (0.59–0.75)	<0.414	73.9 (53.5–87.5)	54.1 (44.3–63.6)
	18-Hydroxycortisol	0.61 (0.47–0.75)	<2.948	43.5 (25.6–63.2)	88.0 (82.5–92.8)
	*Post-ACTH*				
Ipsilateral PA (UU)	Aldosterone	0.89 (0.82–0.96)	>3.352	72.0 (52.4–58.7)	92.0 (85.6–97.8)
	18-Oxocortisol	0.88 (0.80–0.95)	>0.611	76.0 (56.6–88.5)	88.5 (81.3–93.2)
	18-Hydroxycortisol	0.81 (0.72–0.90)	>4.745	76.0 (56.6–88.5)	75.2 (66.5–82.2)
Contralateral PA (UU)	Aldosterone	0.89 (0.83–0.96)	<0.637	88.0 (70.0–95.8)	85.0 (77.2–90.4)
	18-Oxocortisol	0.83 (0.75–0.91)	<0.171	84.0 (60.9–91.1)	76.1 (67.5–83.0)
	18-Hydroxycortisol	0.64 (0.50–0.77)	<2.518	44.0 (26.7–62.9)	86.7 (73.3–91.8)

UU presented unilateral results for both unstimulated and stimulated AVS

*ACTH* adrenocorticotropic hormone, *AUC* area under the curve, *AVS* adrenal venous sampling, *CI* confidence interval, *PA* primary aldosteronism

## Discussion

Our study revealed two major findings. First, we demonstrated that an LC–MS/MS assay has greater utility than the traditional immunoassay in enhancing the assessment of selectivity and discrimination of unilateral PA. Second, we revealed the diagnostic value of the unilateral secretion ratios of aldosterone and the hybrid steroids 18-oxocortisol and 18-hydroxycortisol for PA subtype classification. In addition, we examined the TAIPAI dataset of AVS studies and compared LC–MS/MS assay and immunoassay results for both unstimulated and ACTH-stimulated AVS in patients with PA.

In line with the results of other studies [3, 8, 12], we discovered that multiple alternative steroids (comprising androstenedione, corticosterone, 11-deoxycortisol, 17-hydroxyprogesterone, DHEA, and progestogen) were superior to cortisol in determining selectivity. LC–MS/MS-derived SIs based on cortisol and alternative steroids improved the AVS success rates for unstimulated and stimulated AVS. Furthermore, a large set of cases that were initially regarded as unsuccessful on the basis of immunoassay were confidently determined to be successful because at least two of the LC–MS/MS-derived SIs were higher than the criteria. Our results highlight the advantage of LC–MS/MS over immunoassay in performing simultaneous measurements of multiple steroids in one panel, allowing for the comprehensive confirmation of technical success in AVS.

For lateralization ratios, the LC–MS/MS-derived LI of aldosterone that was corrected by 13 steroids was considerably higher than the immunoassay-derived LI of aldosterone. This finding is reflected in the first set of results obtained in our study, which revealed the differences in aldosterone measurements between immunoassay and LC–MS/MS, and these differences were particularly prominent in the low concentration range. The cross-reactivity of antibodies between aldosterone at low concentrations and other steroids at high concentrations can affect the accuracy of immunoassays in the measurement of aldosterone [2]. Consequently, higher LI values were obtained through LC–MS/MS than through immunoassay, and LC–MS/MS significantly improved the identification of unilateral disease in PA. The discrepancy in LI and lateralization between the two assays was more significant after ACTH stimulation; 20 and 3 patients were determined to have unilateral PA on the basis of LC–MS/MS and immunoassay, respectively. Ma et al. suggested that high cortisol concentrations following ACTH stimulation are beyond the usual detection range of an immunoassay. Thus, cross-reactivity could have biased the results [13].

In contrast to the considerable decrease in the lateralization of the PA assessed by immunoassay, ACTH stimulation had a lesser effect on lateralization status when LC–MS/MS-derived LI was used. Studies have suggested that ACTH stimulation is a double-edged sword because it improves the success rate of cannulation but neutralizes the aldosterone step-up between bilateral adrenals by promoting contralateral aldosterone secretion [1, 14]. However, the surgical cure for PA is dependent on recognizing unilateral sources through AVS. Therefore, LC–MS/MS can enable the use of candidates for adrenalectomy that were restricted by inconclusive AVS when immunoassay was used. In our study, two patients who were initially identified as having bilateral PA on the basis of immunoassay were determined to have unilateral PA when LC–MS/MS was used. Eventually, they achieved complete resolution after undergoing adrenalectomy.

The second key finding of our study is that the secretion ratios of aldosterone, 18-hydroxycortisol, and 18-oxocortisol, as measured using LC–MS/MS, are novel indicators for unilateral PA. Traditionally, LI was the indicator used to assess the aldosterone excess; the assessment involved comparing the aldosterone concentrations of two adrenals. In the present study, we used the secretion ratio of aldosterone to assess aldosterone excess by comparing aldosterone concentrations to total steroid concentrations. These findings can be used to predict lateralization when only unilateral AVS is successful. For unilateral ipsilateral disease, all three steroids (i.e., aldosterone, 18-hydroxycortisol, and 18-oxocortisol) exhibited diagnostic accuracy with AUCs of >0.8, whereas 18-oxocortisol and aldosterone exhibited the highest diagnostic power for unstimulated and stimulated AVS, respectively. For unilateral contralateral disease, only 18-oxocortisol and aldosterone exhibited significant suppression on the contralateral side. This suggested that a sufficient amount of 18-hydroxycortisol was derived from the contralateral zona fasciculata through CYP11B1 when CYP11B2 activity was suppressed [15]. On the basis of our results, we propose the use of the secretion ratio of 18-oxocortisol at pre-ACTH and that of aldosterone at post-ACTH for diagnosing unilateral ipsilateral and contralateral disease, respectively. Another advantage of the aforementioned secretion ratios is that they enable a comprehensive evaluation of the distribution of individual steroid secretion in a single adrenal gland. When we used the cutoffs to predict ipsilateral disease in unstimulated AVS, we discovered that two patients had bilateral vigorous aldosterone excess. The secretion ratios could be used to accurately distinguish between aldosterone excess with a low bilateral secretion level and that with a high bilateral secretion; this could not be achieved with the LI. A study reported clinical and biochemical benefits for patients with BAH after undergoing unilateral adrenalectomy [16]. A comparison of the secretion ratios of aldosterone in bilateral AV may aid clinical decision-making when unilateral adrenalectomy is regarded as a means of attenuating the burden of aldosterone excess in bilateral PA.

In several previous studies, attempts have been made to predict lateralization using one-sided AV samples [17–19]. However, in our study, we essentially considered both pre-ACTH and post-ACTH conditions, whereas previous relevant studies only utilized one sampling method. By considering both conditions, we were able to achieve more robust and conclusive results in terms of lateralization. Furthermore, our utilization of LC–MS/MS led us to discover that 18-hydroxycortisol and 18-oxocortisol also showed promising outcomes.

The secretion ratios of 18-hydroxycortisol and 18-oxocortisol may provide additional information for discriminating PA spectra. Several studies have reiterated the utility of these hybrid steroids in differentiating APA from BAH through the use of urine or serum samples [15, 20, 21]. However, when CYP11B2 immunostaining is implemented, PA is no longer regarded as a binary classification because multiple additional sources of aldosterone excess, such as multifocal APA or aldosterone-producing cell clusters (APCC), may be present in both adrenal glands. Furthermore, a study used imaging mass spectrometry to demonstrate that aldosterone and 18-oxocortisol specifically accumulate in the APCC in adrenal tissue sections [22]. In addition to the histologic spectrum of PA, some patients with unilateral PA exhibit discrepant results for unstimulated and stimulated AVS, reflecting the wide range of disease severity in the PA spectrum [3, 23]. In our study, the secretion ratio of 18-hydroxycortisol among AVs was significantly different for robust unilateral PA (UU) but not for intermediate unilateral PA (UA), suggesting that 18-hydroxycortisol could be a potential index for unilateral PA with a more severe phenotype. In summary, the secretion ratios of hybrid steroids are promising indicators for discriminating the PA spectrum, and further studies are warranted to establish the clinical significance of these discriminating markers.

Few studies have explored the effect of low-grade cortisol concentration in AVS interpretation. A small single-center study, involving patients with both PA and ACS, reported that ACS had a limited effect on AVS interpretation after ACTH stimulation [5]; this finding is consistent with that of the present study, that is, we did not detect any significant difference in selectivity and lateralization between the ACS and non-ACS groups when LC–MS/MS was used. The results pertaining to CT-detectable tumors indicated that a subgroup analysis of the bilateral or unilateral source of autonomous cortisol production should have been conducted, but the small sample size limited our ability to conduct further analysis in the ACS group. The role of ACS in diagnostic PA must be verified through an examination of larger populations.

In addition to the aforementioned limitation regarding ACS subgroup analysis, the present retrospective study has several other limitations. First, this was a single-center study that examined an East Asian cohort; because of differences among ethnic groups, AVS protocols, and interpretation criteria, the present results should be generalized with caution. Second, our analysis of the PA spectrum was limited by the incomplete postoperative follow-up data and the pathologies that were based on hematoxylin and eosin histology instead of immunostaining. Further studies with complete outcome data and CYP11B1/ CYP11B2 immunostaining are required to clarify the PA spectrum link with steroid profiling upstream and the pathologic and clinical outcome downstream.

LC–MS/MS assay optimizes the assessment of selectivity and lateralization in AVS, and it can identify more candidates for curable adrenalectomy than immunoassay can. In addition, the present study is the first to demonstrate the use of the secretion ratios of aldosterone, 18-oxocortisol, and 18-hydroxycortisol in unilateral AV as a discriminating marker for PA lateralization, which can be advantageous when only unilateral AVS is successful. Finally, the present study also verified that steroid profiling is promising for establishing a diagnostic method for the PA of varying levels of severity.

## Supplementary information

Supplementary Methods

## Data Availability

All data generated or analyzed during this study are included in this published article or in the data repositories listed in the references. The supplementary files are available at 10.6084/m9.figshare.19387049.v1.

## References

[1] Rossitto G, Amar L, Azizi M, Riester A, Reincke M, Degenhart C (2020). Subtyping of primary aldosteronism in the AVIS-2 study: assessment of selectivity and lateralization. J Clin Endocrinol Metab.

[2] Eisenhofer G, Dekkers T, Peitzsch M, Dietz AS, Bidlingmaier M, Treitl M (2016). Mass spectrometry-based adrenal and peripheral venous steroid profiling for subtyping primary aldosteronism. Clin Chem.

[3] Turcu AF, Wannachalee T, Tsodikov A, Nanba AT, Ren J, Shields JJ (2020). Comprehensive analysis of steroid biomarkers for guiding primary aldosteronism subtyping. Hypertension.

[4] Wu VC, Hu YH, Er LK, Yen RF, Chang CH, Chang YL (2017). Case detection and diagnosis of primary aldosteronism – The consensus of Taiwan Society of Aldosteronism. J Formos Med Assoc.

[5] O’Toole SM, Sze WC, Chung TT, Akker SA, Druce MR, Waterhouse M (2020). Low-grade cortisol cosecretion has limited impact on ACTH-stimulated AVS parameters in primary aldosteronism. J Clin Endocrinol Metab.

[6] Fassnacht M, Arlt W, Bancos I, Dralle H, Newell-Price J, Sahdev A (2016). Management of adrenal incidentalomas: European Society of Endocrinology Clinical Practice Guideline in collaboration with the European Network for the Study of Adrenal Tumors. Eur J Endocrinol.

[7] Peng KY, Liao HW, Chan CK, Lin WC, Yang SY, Tsai YC (2020). Presence of subclinical hypercortisolism in clinical aldosterone-producing adenomas predicts lower clinical success. Hypertension.

[8] Peitzsch M, Dekkers T, Haase M, Sweep FC, Quack I, Antoch G (2015). An LC-MS/MS method for steroid profiling during adrenal venous sampling for investigation of primary aldosteronism. J Steroid Biochem Mol Biol.

[9] Chang YL, Chen GY, Lee BC, Chen PT, Liu KL, Chang CC, et al. Data supplement for “LC-MS/MS optimizing adrenal vein sampling interpretation and the novel indicators for primary aldosteronism lateralization: the secretion ratios of aldosterone, 18-oxocortisol, and 18-hydroxycortisol”. Figshare. 2022. 10.6084/m9.figshare.19387049.v1

[10] Williams TA, Lenders JWM, Mulatero P, Burrello J, Rottenkolber M, Adolf C (2017). Outcomes after adrenalectomy for unilateral primary aldosteronism: an international consensus on outcome measures and analysis of remission rates in an international cohort. Lancet Diabetes Endocrinol.

[11] Xing Y, Edwards MA, Ahlem C, Kennedy M, Cohen A, Gomez-Sanchez CE (2011). The effects of ACTH on steroid metabolomic profiles in human adrenal cells. J Endocrinol.

[12] Ceolotto G, Antonelli G, Maiolino G, Cesari M, Rossitto G, Bisogni V (2017). Androstenedione and 17-α-hydroxyprogesterone are better indicators of adrenal vein sampling selectivity than cortisol. Hypertension.

[13] Ma Y, Chen H, Chen F, Jiang J, Guo W, Li X, et al. Mass spectrometry-based cortisol profiling during adrenal venous sampling reveals misdiagnosis for subtyping primary aldosteronism. Clin Endocrinol (Oxf). 2022;96:680–9. 10.1111/cen.14666 34970750

[14] El Ghorayeb N, Mazzuco TL, Bourdeau I, Mailhot JP, Zhu PS, Thérasse E (2016). Basal and post-ACTH aldosterone and its ratios are useful during adrenal vein sampling in primary aldosteronism. J Clin Endocrinol Metab.

[15] Satoh F, Morimoto R, Ono Y, Iwakura Y, Omata K, Kudo M (2015). Measurement of peripheral plasma 18-oxocortisol can discriminate unilateral adenoma from bilateral diseases in patients with primary aldosteronism. Hypertension.

[16] Sukor N, Gordon RD, Ku YK, Jones M, Stowasser M (2009). Role of unilateral adrenalectomy in bilateral primary aldosteronism: a 22-year single center experience. J Clin Endocrinol Metab.

[17] Barbar B, Truran P, Ramsingh J, Bliss R, Boot C, Ramzan M (2023). Subtyping primary aldosteronism by inconclusive adrenal vein sampling. Clin Endocrinol (Oxf).

[18] Suntornlohanakul O, Soonthornpun S, Srisintorn W, Murray RD, Kietsiriroje N (2020). Performance of the unilateral AV/IVC index in primary hyperaldosteronism subtype prediction: a validation study in a single tertiary centre. Clin Endocrinol (Oxf).

[19] Strajina V, Al-Hilli Z, Andrews JC, Bancos I, Thompson GB, Farley DR (2018). Primary aldosteronism: making sense of partial data sets from failed adrenal venous sampling-suppression of adrenal aldosterone production can be used in clinical decision making. Surgery.

[20] Mulatero P, di Cella SM, Monticone S, Schiavone D, Manzo M, Mengozzi G (2012). 18-Hydroxycorticosterone, 18-hydroxycortisol, and 18-oxocortisol in the diagnosis of primary aldosteronism and its subtypes. J Clin Endocrinol Metab.

[21] Nakamura Y, Satoh F, Morimoto R, Kudo M, Takase K, Gomez-Sanchez CE (2011). 18-Oxocortisol measurement in adrenal vein sampling as a biomarker for subclassifying primary aldosteronism. J Clin Endocrinol Metab.

[22] Sugiura Y, Takeo E, Shimma S, Yokota M, Higashi T, Seki T (2018). Aldosterone and 18-oxocortisol coaccumulation in aldosterone-producing lesions. Hypertension.

[23] Wannachalee T, Zhao L, Nanba K, Nanba AT, Shields JJ, Rainey WE (2019). Three discrete patterns of primary aldosteronism lateralization in response to cosyntropin during adrenal vein sampling. J Clin Endocrinol Metab.

