# Supplementary material for: Optimizing adrenal vein sampling in primary aldosteronism subtyping through LC–MS/MS and secretion ratios of aldosterone, 18-oxocortisol, and 18-hydroxycortisol
Source: Hypertens Res. 2023 Jun 13;46(8):1983–94. doi: 10.1038/s41440-023-01347-2 (PMC10404510; doi:10.1038/s41440-023-01347-2)
Supplement: Supplementary file 1 — Supplementary Methods [file 41440_2023_1347_MOESM1_ESM.pdf]

Supplemental Methods

Specimen processing

100 µl serum samples were aliquoted and mixed well with 100 µL 0.1M ZnSO4 solution, followed by the addition of 800 µL deionized water and internal standard solutions prior to solid phase extraction (SPE). 96-well Sep-Pak 40 mg C18 SPE cartridges (Waters, Milford, MA) were employed and conditioned with 1 mL MeOH followed by 1 mL DI water with 96-well SPE manifold under vacuum. The resulting sample mixtures were then loaded onto conditioned cartridges carefully and washed with 2 mL 30% MeOH. Steroids were eluted with 2 mL MeOH and dried with stream nitrogen. The dried residues were reconstituted with 100 µl MeOH prior to LC-MS/MS analysis.

LC-MS/MS procedure

An Agilent UHPLC 1290 (Santa Clara, CA) coupled to an AB Sciex QTRAP 6500 (Foster City, CA) is employed to perform LC-MS/MS analysis with multiple reaction monitoring (MRM) mode. A 10 µL sample of samples were injected onto a Biphenyl column (2.1x 100 mm, 2.6 µm) (Phenomenex, Torrance, CA) and the analytical column was maintained at 40 ˚C. The mobile phases were composed of solvent A: water/0.1% formic acid and solvent B: MeOH/0.1% formic acid. The gradient elution program will initially be as the following: 0–1 min: 35% B; 1-1.5 min: linear gradient from 35 to 70% B; 1.5-5 min: linear gradient from 70 to 95% B, and keep in 95% B for 3 min. The flow rate will be 400 µL min-1. The mass spectrometer was operated in positive ionization mode with the ion spray voltage set to 5500 V, nebulizer (gas 1) pressure set to 50 psi, drying gas (gas 2) pressure set to 60 psi, and gas temperature set to 600°C. The curtain gas pressure was 30 psi. The scheduled multiple reaction monitoring (sMRM) method contained at least two transitions for each analyte and ISTD. The total cycle time in sMRM mode was set to 0.8 sec. The MRM transitions are listed in Table S1. MRM results were processed with Skyline (21.1.0.278).

Supplemental Tables

Table S1 MRM transitions and retention time (RT) for monitored steroid hormones

Steroid	RT (min)	Q1	Q3	DP (V)	EP (V)	CE (V)	CXP (V)
Androstenedione	3.8	287	97	100	10	30	10
			109	100	10	30	10
			173	100	10	30	10
Aldosterone	3.5	361.2	315	80	10	25	35
			299	80	10	30	10
			279	80	10	30	10
Cortisol	3.9	363	121	90	10	30	10
			269	90	10	25	10
			241	90	10	30	20
DHEA	4.1	289.4	213	60	10	25	10
			197	60	10	25	20
			157	60	10	35	10
DHEAS	3.7	369.2	253	60	10	20	15
			213	60	10	30	15
			197	60	10	35	15
Corticosterone	3.9	347.3	121	100	10	30	8
			171	100	10	30	9
			175	100	10	30	8
11-Deoxycortisol	3.7	347.3	97	100	12	30	10
			109	100	12	30	10
			269	100	12	25	10
21-Deoxycortisol	3.2	347.3	269	100	10	25	25
			121	100	10	30	10
			175	100	10	25	20
11-Deoxycorticosterone	4.7	331.3	97	110	12	25	10
			109	110	12	30	10
			123	110	12	30	15
17-Hydroxyprogesterone	4.2	331.3	97	100	9	25	10
			271	100	9	25	20
			109	100	9	35	10
Cortisone	3.2	361.2	163	120	15	35	10
			121	120	15	35	10
			299	120	15	25	25
Testosterone	4.1	289.3	97	100	10	25	15
			109	100	10	25	15
			253	100	10	25	25
Progesterone	5.4	315.3	97	100	10	25	10
			109	100	10	30	10
			122.8	100	10	30	15
21-Deoxycortisol	3.2	355.4	180	100	8	25	10
			125	100	8	25	10

18-Hydroxycortisol	2.7	379	267	80	8	25	20
			285	80	8	25	25
18-Oxocortisol	2.9	377	313	100	8	25	25
			295	100	8	30	25
Tetrahydrocorticosterone	3.3	351	159	20	10	45	10
			145	20	10	40	10
Tetrahydrocortisol	4	367	185	30	14	35	25
			175	30	14	40	15
Progesterone_d9	5.4	324	100	100	10	25	15
Testosterone_d3	4.1	292	97	100	10	25	15
Cortisol_d4	3	367.4	121	90	9	30	10
			271	90	9	25	10
DHEA_d5	4.1	294.2	258	60	10	15	25
			218	60	10	25	15
17-Hydroxyprogesterone_d8	4.2	339.4	100	100	10	30	10
			113	100	10	35	10

RT, retention time; MRM, multiple reaction monitoring; DP, declustering potential; EP, entrance potential; CE, collision energy; CXP, collision cell exit potential

Supplemental Figures

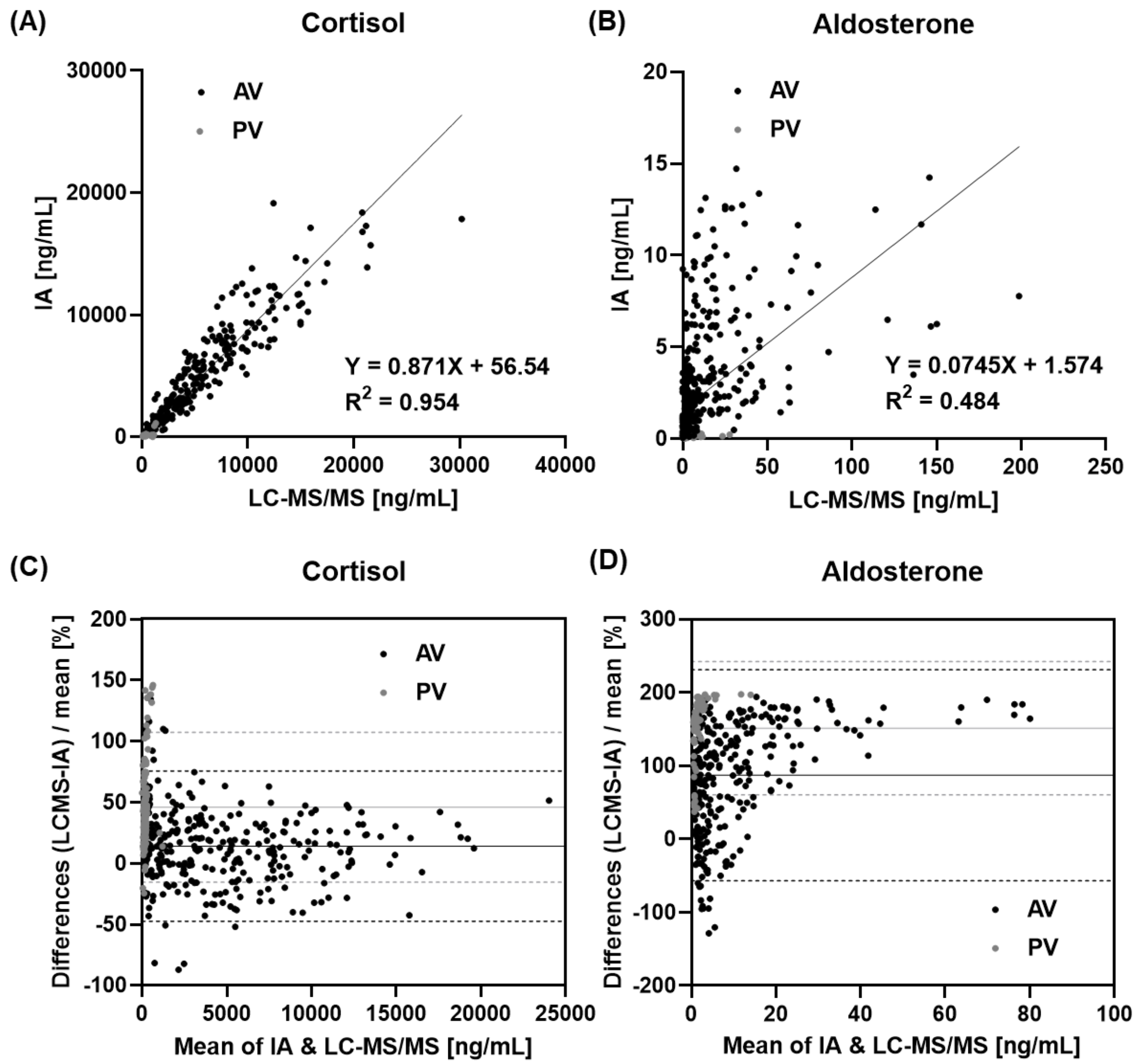

Figure S1. Comparison of LC-MS/MS and immunoassays results. Deming regression comparison between LC-MS/MS and immunoassays measured (A) cortisol and (B) aldosterone in plasma samples of bilateral adrenal veins (black dot) and peripheral vein (grey dot). Bland-Altman plots for (C) cortisol and (D) aldosterone assays. Deming regressions coefficient presented as R^2. The mean differences of adrenal veins samples and peripheral vein are shown as black and grey lines, while the 95% limits of agreement (LOAs) are depicted as dashed black and grey lines, respectively.

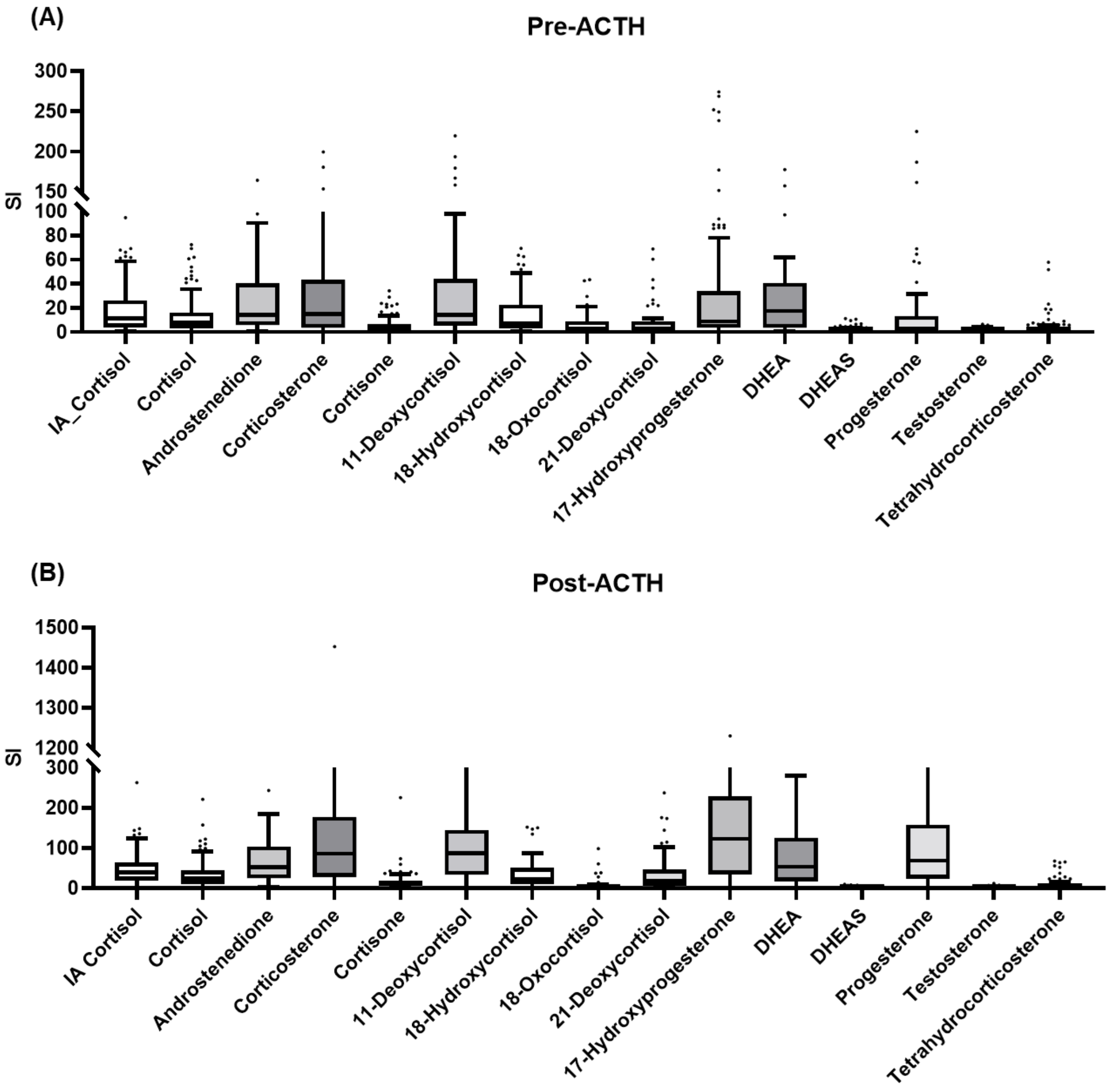

Figure S2. Tukey box-whisker plots comparison of selectivity index using immunoassay cortisol and LS-MS/MS steroids before and after ACTH stimulation in patient with primary aldosteronism. ACTH, adrenocorticotrophic hormone; IA, immunoassay; SI, selectivity index; PA, primary aldosteronism.
